# New Eye Cleansing Product Improves Makeup-Related Ocular Problems

**DOI:** 10.1155/2015/831628

**Published:** 2015-08-11

**Authors:** Masako Okura, Motoko Kawashima, Mikiyuki Katagiri, Takuji Shirasawa, Kazuo Tsubota

**Affiliations:** ^1^Department of Aging Control Medicine, Juntendo University Graduate School of Medicine, 3-1-3 Hongo, Bunkyo-ku, Tokyo 113-8431, Japan; ^2^Eye Clinic Tenjin, 2-11-1 Tenjin, Chuo-ku, Fukuoka 8100001, Japan; ^3^Department of Ophthalmology, Keio University School of Medicine, 35 Shinanomachi, Shinjuku-ku, Tokyo 1608582, Japan

## Abstract

*Purpose*. This study evaluated the effects of using a newly developed eye cleansing formulation (Eye Shampoo) to cleanse the eyelids for 4 weeks in a parallel-group comparative study in women with chronic eye discomfort caused by heavy use of eye makeup and poor eye hygiene habits. *Methods*. Twenty women participants who met the inclusion criteria were randomly allocated to 2 groups comprising 10 participants each. The participants were asked to use either artificial tears alone or artificial tears in conjunction with Eye Shampoo for 4 weeks. The participants answered the questionnaire again and were reexamined, and changes in symptoms within each group and variations of symptoms between the two groups were statistically analyzed. *Results*. In the group using only artificial tears, improvements in subjective symptoms but not in ophthalmologic examination results were found. In the group using Eye Shampoo together with artificial tears, significant improvements were observed in the subjective symptoms, meibomian orifice obstruction, meibum secretion, tear breakup time, and superficial punctate keratopathy. *Conclusion*. In patients with chronic eye discomfort thought to be caused by heavy eye makeup, maintaining eyelid hygiene using Eye Shampoo caused a marked improvement in meibomian gland blockage and dry eye symptoms.

## 1. Introduction

In recent years, a large number of cosmetics for the eye have become popular among young women. A large number of patients have reported various symptoms resulting from the use of such cosmetic products including oil-based makeup removers. These products also appear to increase the risk of meibomian gland dysfunction (MGD) in young people.

**Figure 1:**
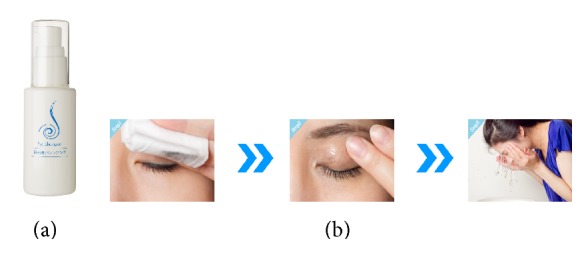
Lid hygiene technique. (a) Eye Shampoo (http://www.eyeshampoo.com/). (b) Lid cleansing using Eye Shampoo.

Eyelid hygiene has been reported to be an effective technique for managing MGD [1–3]. Several lid scrubs including OCuSOFT (OCuSOFT Corp., Rosenberg, Texas) are available commercially, and reports of their effectiveness have been published [3–7]. A hypoallergenic makeup removal product that can also be used for treatment of MGD was recently developed in Japan. This product, Eye Shampoo (MediProduct Co., Ltd., Tokyo, Japan), is a nonirritating eye cleansing formulation with a pH of 7.4, an osmolarity of 300 mOsm/L, and ingredients with anti-inflammatory (dipotassium glycyrrhizate) and moisturizing (sodium hyaluronate) properties (Figure 1(a)).

In this study, we investigated the effectiveness of eyelid hygiene using Eye Shampoo for young women who routinely wore heavy makeup.

## 2. Methods

### 2.1. Subjects

Thirty women between 20 and 40 years of age were recruited. They currently use all the following makeup tools: foundation, eyelash makeup tools such as mascara, false eyelashes and eyelash extensions, eye shadow, eyeliner with inside liner, and eye makeup remover. Inclusion criteria included subjective symptoms of chronic eye discomfort and meibomian gland blockage. Individuals were excluded if they had currently undergoing treatment of ocular surface diseases including blepharitis, dry eye disease, and Sjögren syndrome. Of the 30 women recruited, 29 gave informed consent to participate in the study. Each participant underwent screening for eligibility, received written and oral information about the study, and provided written consent to participate. Each also completed a background survey with questions including contact lens wear, history of laser in situ keratomileusis, eye-washing habits, history of chalazion, and use of eye drops. Screening examinations of the 29 volunteers were conducted, and 20 study participants who met the criteria were selected (mean age ± range, 28.1 ± 5.7 years) (Table 1).

This study followed the tenets of the Declaration of Helsinki, and the protocol was approved prospectively by the Shirasawa Clinical Research Center Ethical Review Board, Gunma, Japan.

### 2.2. Experimental Protocols

After consistency in ages and primary endpoints had been ensured, the patients were randomly allocated to either the control group, using only artificial tears (*n* = 10), or the Eye Shampoo group, using Eye Shampoo in conjunction with artificial tears (*n* = 10). Test products were sent by mail to the participants of each group and were used for 4 weeks, after which the examinations were repeated. Participants were allowed to use artificial tears up to 6 times per day.

The participants answered a study questionnaire and underwent eyelid margin and ocular surface examinations, including tear breakup time (BUT) and fluorescein staining score. Bacterial cultures were taken from the lid margin. They were also instructed to self-record their lid hygiene performing status and any adverse events in a study diary throughout the intervention period. Primary outcomes included subjective symptoms, meibomian orifice obstruction, and meibum secretion evaluated by compression.

### 2.3. Eyelid Hygiene Using Eye Shampoo

Study participants in the Eye Shampoo group used eye makeup remover and face-washing soap after awakening and before going to bed. They were asked to add Eye Shampoo to this hygiene regimen by pumping the Eye Shampoo onto the hand or a piece of cotton, gently spreading it around the eyes, lightly massaging it to remove any impurities at the root of the eyelashes, and washing it away (Figure 1(b)). They were allowed to use a cotton swab if they were still concerned about impurities. They were asked to place a drop of artificial tears in each eye after the eye-washing procedure. Participants in the control group followed the same hygiene regimen with the exception of the Eye Shampoo.

### 2.4. Subjective Symptom Questionnaire

The participants were asked to answer questions regarding 15 symptoms (Table 2). Responses of “none,” “mild,” “moderate,” and “severe” were scored from 0 to 3, respectively. The totals were calculated and compared among the 15 symptoms.

### 2.5. Eyelid Margin and Ocular Surface Examinations

Meibomian orifice obstruction and decreased meibum secretion were classified into 3 stages, scored as 0, 1, or 2. Decreased meibum secretion was evaluated with a 2-second duration of extrusion of the glands of the upper eyelid using the thumb. Meibomian gland orifice abnormalities (telangiectasia, displacement of the mucocutaneous junction, and eyelid margin abnormalities), BUT, superficial punctate keratopathy (SPK), eyelid culture, strip meniscometry, and measurement of eyelash length were also evaluated. Meibomian gland orifice abnormalities and SPK were classified into 3 stages, scored as 0, 1, or 2. Numerical values were indicated using mean ± standard deviation.

### 2.6. Statistical Analysis

Measurements for strip meniscometry, BUT, and eyelash length were analyzed using the *t*-test. For the parameters evaluated using three or four grades, intragroup comparisons were carried out and analyzed using the Wilcoxon signed rank test. Variations in the intergroup comparisons were evaluated with the Mann-Whitney *U* test. Significance was assumed to be *P* < 0.05.

## 3. Results

All participants completed the study, and results from all were included in the analysis. The lowest use rate among the participants was 62%.

### 3.1. Subjective Symptoms

**Figure 2:**
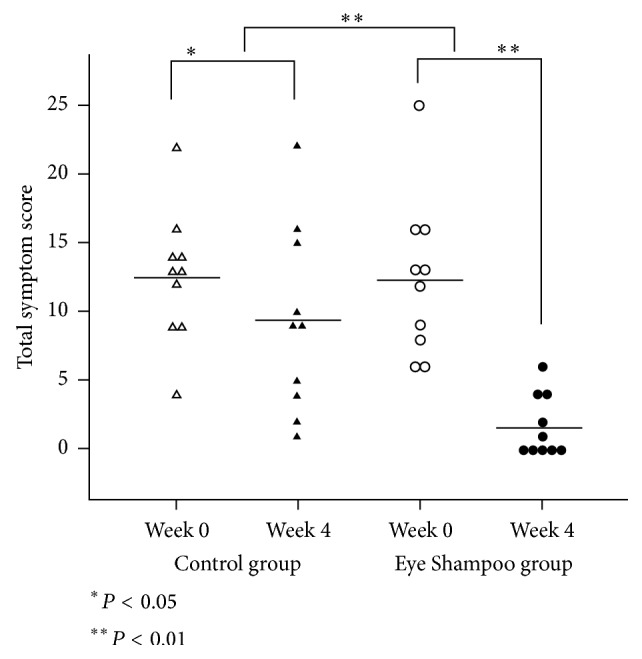
Subjective symptom scores for the two groups.

**Figure 3:**
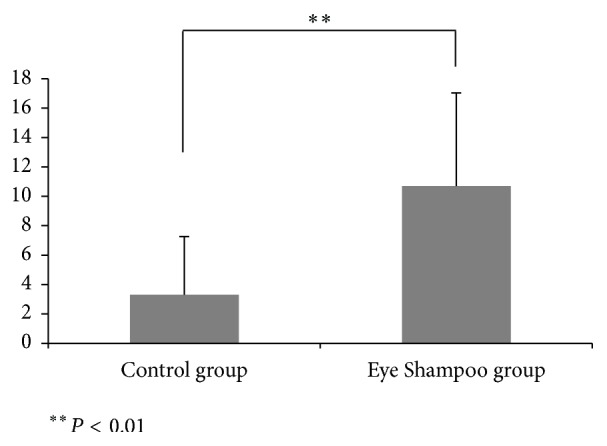
Variation in subjective symptom scores between the two groups.

Both groups exhibited significant improvement in subjective symptoms (control group, *P* = 0.034; Eye Shampoo group, *P* = 0.005) (Table 3, Figure 2). In the intergroup comparison of variations, scores in the Eye Shampoo group were significantly higher than those in the control group (*P* = 0.005) (Figure 3).

### 3.2. Eyelid Margin Examination

**Figure 4:**
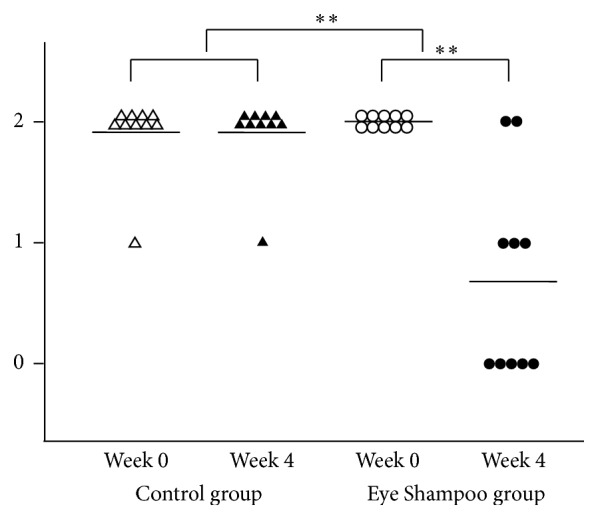
Meibomian gland orifice obstruction in the two groups. 0, absent; 1, slight; 2, prominent.

**Figure 5:**
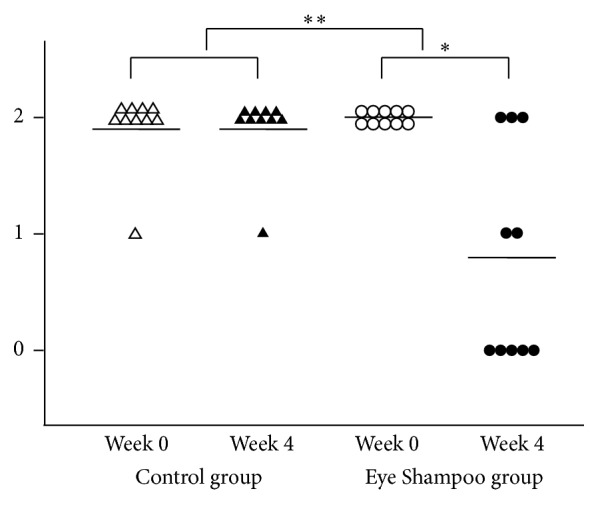
Meibum secretion in the two groups, evaluated by pressing the middle of the upper eyelid with a thumb. 0, expression with mild pressure; 1, expression with moderate pressure; 2, no expression with moderate pressure.

**Figure 6:**
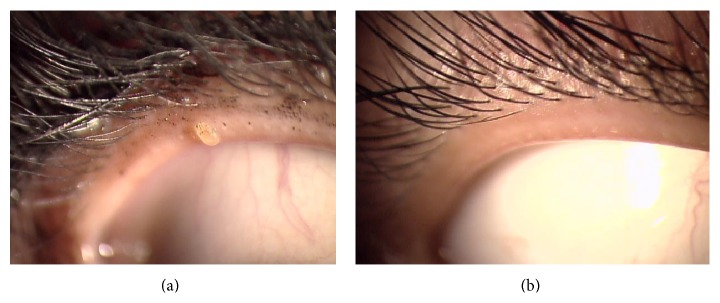
Plugging in the upper eyelid, with improvement after 4 weeks of intervention. (a) Week 0, with mascara and glue adhering to the eyelashes. (b) Week 4, after using artificial tears and Eye Shampoo.

**Figure 7:**
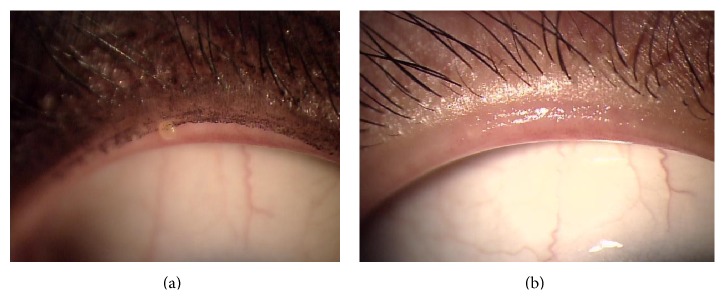
Tapioca sign in the upper eyelid, with improvement after 4 weeks of intervention. (a) Week 0, adhering to eyeliner inside eyelid margin. (b) Week 4, after using artificial tears and Eye Shampoo.

**Figure 8:**
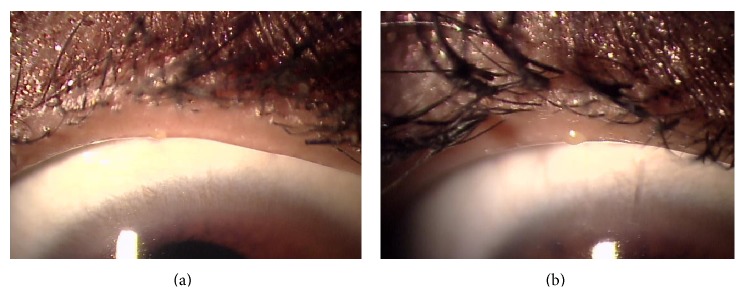
Tapioca sign in the upper eyelid, with no improvement after 4 weeks; eyelash extensions are present in both images. (a) Week 0. (b) Week 4, after using artificial tears.

The Eye Shampoo group exhibited significant improvement in meibomian orifice obstruction scores (*P* = 0.009); scores were also higher in the Eye Shampoo group than in the control group (*P* = 0.001) (Figure 4). The Eye Shampoo group showed significant improvement in meibum secretion scores as well (*P* = 0.002) (Table 3, Figure 5). Abnormal findings around the orifices (vascular engorgement, anterior or posterior replacement, and irregular eyelid margin) were not significantly different in either group. Two images (Figures 6 and 7) show marked effects seen in the Eye Shampoo group, and one image (Figure 8) shows a typical presentation in the control group.

### 3.3. Ocular Surface Examination

No significant variation of BUT was recognized within the control group. However, BUT was significantly extended within the Eye Shampoo group and was also significantly higher in the Eye Shampoo group than in the control group (right eye: intragroup, *P* = 0.008; intergroup, *P* = 0.002; left eye: intragroup, *P* = 0.004; intergroup, *P* = 0.002). No change in SPK was observed within the control group. However, both intragroup and intergroup variations in SPK were significantly higher in the Eye Shampoo group (intragroup, *P* = 0.025; intergroup, *P* = 0.007) (Table 4).

### 3.4. Other Examinations

There was no significant difference in strip meniscometry scores between the groups. However, in the intragroup comparisons, scores within the Eye Shampoo group were significantly improved after intervention (right eye, *P* = 0.002; left eye, *P* = 0.014).

Before intervention, resident microbiota was detected by bacterial culture in 3 participants in each of the two groups. After 4 weeks, however, resident microbiota was detected in 3 participants in the control group but in no participants in the Eye Shampoo group.

A questionnaire regarding subjective symptoms was administered to the participants at the conclusion of the study. We asked participants if they had become aware of “stinging,” “blurred by oil,” “feeling pain,” “experiencing itchiness,” and other symptoms after using eye makeup removers. Eight participants in the control group responded with “blurred by oil,” 2 responded with “stinging,” and 1 responded with “foreign body sensation.” In the Eye Shampoo group, no participants reported symptoms of “blurriness,” “stinging,” “itchiness,” and so forth. However, 3 participants responded with “felt refreshed” and others responded favorably with comments such as “felt safe and was able to completely remove make up,” “did not feel any stinging even though I scrubbed,” and “knew that I had missed spots even after washing my face.”

## 4. Discussion

Our results indicate that eyelid hygiene using Eye Shampoo markedly improved objective ocular surface findings, symptoms of dry eye, and chronic eye discomfort thought to be caused by the heavy use of eye makeup. In the group that used artificial tears in conjunction with Eye Shampoo, significant differences from the control group were observed in subjective symptoms, orifice obstruction, decreased meibum secretion by pressure application, and the examinations for BUT and SPK. A significant improvement in the volume of pooled tears (measured by strip meniscometry) was also noted in the Eye Shampoo group.

In the case of obstructive MGD, tear film abnormalities such as a decreased lipid layer thickness are directly caused by a decrease in meibum secretion. It has been reported that the tear film may become unstable because of the thinning of the lipid layer, which is reflected by short BUT. It has also been reported that the tear film in the lower cornea breaks up easily, and in that area, a corresponding corneal epithelium disorder may be observed [8]. The treatment goal is to improve abnormalities observable with a slit lamp, such as the short BUT and the resulting epithelial damage [9]. The site of decreased tear film stability is likely to be contaminated with eye makeup, thereby shortening BUT and causing SPK. In this study, we found that implementation of eyelid hygiene using Eye Shampoo helped minimize areas missed by eye makeup removal and kept the eyelid clean. This, in turn, led to reduction in obstruction, improvement of meibum secretion measured by pressure application, stabilization of tear film, improvement in subjective symptoms, increase in BUT, and improvement in SPK. It is also possible that this led to improved strip meniscometry measurements of the volume of pooled tears in the tear meniscus.

In the present study, no adverse events were reported and good results were obtained using Eye Shampoo. Moreover, no participants reported irritation using Eye Shampoo, an advantage of continuing lid hygiene. In addition, participants reported that Eye Shampoo was easily available and that they felt that their eyelashes were healthier. These suggest that continued use of Eye Shampoo could have additional favorable effects on the eyelashes; however, there are issues to be addressed, including no sample size calculation at the start of the study, the small sample size, and the fact that only young adults were included. Further investigation is warranted.

In recent years, “eye appeal makeup” has become popular among young people in Japan, and some have presented with dry eye symptoms due to obstructed meibomian glands at a young age. The most likely cause is an eye makeup technique called “inside liner,” which is eyeliner that is applied to the eyelid margins over the meibomian glands so that the eyes will appear to be larger. Another cause may be that eyeliner, eye shadow, mascara, and foundation become mixed with sweat and tears over time, and the meibomian glands may become obstructed by the oils in the eye makeup. A thin, oily film mixed with pigments may cover the base of the eyelashes over the meibomian glands. Eye makeup may also migrate to the ocular surface through blinking or using eye drops [10, 11]. Kawakita et al. reported that the ocular surface migration rate (96%) and contamination scores of makeup applied to the base of the eyelashes increased when eye drops were also used [13]. We have encountered cases in which gel-like eyeliner that flaked off created a foreign body that remained in the conjunctiva. Furthermore, eyelash extensions must be used with care, as they may cause the area around the eyelashes, including the meibomian glands, to become contaminated. Some of the patients in this study presented with artificial entropion, which occurred as false eyelashes pressed the natural eyelashes down. Moreover, it appears that eyelid glue used to create a fold not only accelerates tear evaporation as it obstructs closure of the eyelid but also affects meibomian gland secretion. Blinking induces the meibomian glands to produce tears slowly from the eyelid margins. Lacrimal flow associated with blinking, which has been likened to pleated drapes [12], may thereby be affected. We have observed cases in which eyelid glue became twisted and entangled in the eyelashes over time, producing a dandruff-like appearance. It may be necessary to differentiate this presentation from that of* Demodex* [13–15], which, incidentally, we suspected in one of our study participants, although we did not detect* Demodex* upon examination with a speculum.

With cosmetics contaminating the eyes, tear film stability is further decreased, likely generating a vicious cycle. Our results suggest that, by regularly implementing eyelid hygiene, the symptoms of MGD could be improved relatively easily when eye makeup obstructs the meibomian glands in young people. There has been a recent tendency to overwork the eyes from a young age with eye makeup and other products. In this regard, implementation of daily eyelid hygiene to keep the eyelids clean could slow the aging of the eyes.

In conclusion, eyelid hygiene is an effective management technique for meibomian gland and ocular surface health.

## Figures and Tables

**Table 1:** Participants' demographic characteristics.

	Eye Shampoo group	Control group
*N*	10	10
Age (range), years	26.5 (21–33)	29.7 (20–39)
Contact lens use (yes : no)	8 : 2	7 : 3

**Table 2:** Ocular symptoms questionnaire. Data were obtained before and 4 weeks after intervention.

	None	Mild	Moderate	Severe
Eye discomfort (feeling vague discomfort in eyes)	□	□	□	□
Feeling of dryness	□	□	□	□
Foreign body sensation	□	□	□	□
Bleary eyes	□	□	□	□
Gritty feeling	□	□	□	□
Burning sensation	□	□	□	□
Sore eyes	□	□	□	□
Hot	□	□	□	□
Eye discharge (gum in eyes)	□	□	□	□
Stringy mucus	□	□	□	□
Blurriness	□	□	□	□
Eyestrain	□	□	□	□
Eye pain	□	□	□	□
Lacrimation (excessive watering of the eye)	□	□	□	□
Itchiness	□	□	□	□
Red eye	□	□	□	□
Heaviness	□	□	□	□
Extra sensitivity to light	□	□	□	□
Excessive frequent eye blinking (frequent blinks)	□	□	□	□

**Table 3:** Comparison of symptoms, orifice obstruction, and decreased meibum secretion between the groups.

Group/task	Baseline	4 weeks		Variation	
	Mean ± SD	Mean ± SD	*P* (versus baseline)	Mean ± SD	*P* (between-group)
Subjective symptoms					
Control group	12.6 ± 4.8	9.3 ± 6.7	0.034^*∗*^	−3.3 ± 4.0	0.005^*∗∗*^
Eye Shampoo group	12.4 ± 5.8	1.7 ± 2.2	0.005^*∗∗*^	−10.7 ± 6.3	
Orifice obstruction					
Control group	1.9 ± 0.3	1.9 ± 0.3	1.000	0.0 ± 0.0	0.001^*∗∗*^
Eye Shampoo group	2.0 ± 0.0	0.7 ± 0.8	0.009^*∗∗*^	−1.3 ± 0.8	
Decreased meibomian secretion					
Control group	1.9 ± 0.3	1.9 ± 0.3	1.000	0.0 ± 0.0	0.002^*∗∗*^
Eye Shampoo group	2.0 ± 0.0	0.8 ± 0.9	0.014^*∗*^	−1.2 ± 0.9	

^*∗*^ *P* < 0.05, ^*∗∗*^ *P* < 0.01.

**Table 4:** Comparison of strip meniscometry, tear film breakup time (BUT), and superficial punctate keratopathy (SPK) between the groups. SD, standard deviation.

Group/task	Baseline	4 weeks		Variation	
	Mean ± SD	Mean ± SD	*P* (versus baseline)	Mean ± SD	*P* (between-group)
Strip meniscometry (R)					
Control group	5.3 ± 3.6	5.1 ± 3.8	0.900	−0.2 ± 4.9	0.210
Eye Shampoo group	3.9 ± 2.0	5.8 ± 1.9	0.002^*∗∗*^	1.9 ± 1.4	
Strip meniscometry (L)					
Control group	5.2 ± 3.8	5.6 ± 4.0	0.790	0.4 ± 4.6	0.283
Eye Shampoo group	4.1 ± 2.2	6.3 ± 3.3	0.014^*∗∗*^	2.2 ± 2.3	
BUT (R)					
Control group	3.7 ± 1.1	3.2 ± 1.4	0.096	−0.5 ± 0.8	0.002^*∗∗*^
Eye Shampoo group	3.7 ± 1.3	4.7 ± 1.6	0.008^*∗*^	1.0 ± 0.9	
BUT (L)					
Control group	3.7 ± 1.1	3.3 ± 1.4	0.168	−0.4 ± 0.8	0.002^*∗∗*^
Eye Shampoo group	3.7 ± 1.3	4.6 ± 1.4	0.004^*∗∗*^	0.9 ± 0.7	
SPK					
Control group	0.7 ± 0.5	0.9 ± 0.3	0.157	0.2 ± 0.4	0.007^*∗∗*^
Eye Shampoo group	0.6 ± 0.7	0.1 ± 0.3	0.025^*∗*^	−0.5 ± 0.5	

^*∗*^ *P* < 0.05, ^*∗∗*^ *P* < 0.01.
